# Modulation of magnesium deficiency-induced anxiety and HPA axis dysregulation by therapeutic drug treatment

**DOI:** 10.1186/1471-2210-11-S2-A40

**Published:** 2011-09-05

**Authors:** Simone B Sartori, Nigel Whittle, Alfred Hetzenauer, Nicolas Singewald

**Affiliations:** 1 Department of Pharmacology and Toxicology, Institute of Pharmacy, and Center for Molecular Biosciences Innsbruck (CMBI), University of Innsbruck, 6020 Innsbruck, Austria

## Background

Preclinical and some clinical studies suggest a relationship between perturbation in magnesium homeostasis and pathological anxiety, although the underlying mechanisms remain largely unknown. Since there is evidence that Mg^2+^ modulates the hypothalamic-pituitary-adrenal (HPA) axis, we tested whether enhanced anxiety-like behaviour can be reliably elicited by dietary Mg^2+^ restriction and whether Mg^2+^ deficiency is associated with altered HPA axis function.

## Methods

Mice assigned to Mg^2+^-deficient groups were allowed to freely access a 0.005% Mg^2+^-containing diet while control mice were fed a normal, 0.2% Mg^2+^-containing diet. The emotional behaviour of Mg^2+^-deficient mice was assessed in a battery of anxiety tests including the open field test, the light/dark test, the stress-induced hypothermia test, and the hyponeophagia test. Markers of HPA axis function including CRH gene expression and plasma ACTH levels were quantified. Neuronal activation patterns in the HPA system were investigated using mapping of the immediate early gene c-Fos as a marker of neuronal activation in response to an anxiety-provoking situation.

## Results

Compared to controls, Mg^2+^-deficient mice did indeed display enhanced anxiety-related behaviour in numerous anxiety tests. The enhanced anxiety-related behaviour of Mg^2+^-deficient mice was sensitive to chronic desipramine treatment in the hyponeophagia test and to acute diazepam treatment in the open arm exposure test. Mg^2+^ deficiency caused an increase in the transcription of corticotropin releasing hormone in the paraventricular hypothalamic nucleus (PVN), which coincided with elevated ACTH plasma levels, pointing to an enhanced set-point of the HPA axis. Chronic treatment with desipramine reversed the identified abnormalities of the stress axis. Functional mapping of neuronal activity revealed hyper-excitability in the PVN of anxious Mg^2+^-deficient mice and its normalisation through diazepam treatment.

## Conclusions

Overall, the present findings demonstrate the robustness and validity of the Mg^2+^ deficiency model as a mouse model of enhanced anxiety, showing sensitivity to treatment with anxiolytics and antidepressants. It is further suggested that dysregulations in the HPA axis may contribute to the hyper-emotionality in response to dietary induced hypomagnesaemia.

